# Health progression for Covid-19 survivors hospitalized in geriatric clinics in Sweden

**DOI:** 10.1371/journal.pone.0283344

**Published:** 2023-03-22

**Authors:** Laura Kananen, Xu Hong, Martin Annetorp, Jonathan K. L. Mak, Juulia Jylhävä, Maria Eriksdotter, Sara Hägg, Dorota Religa

**Affiliations:** 1 Department of Medical Epidemiology and Biostatistics, Karolinska Institute, Stockholm, Sweden; 2 Faculty of Social Sciences (Health Sciences), Gerontology Research Center, Tampere University, Tampere, Finland; 3 Faculty of Medicine and Health Technology, Tampere University, Tampere, Finland; 4 Division of Clinical Geriatrics, Department of Neurobiology, Care Sciences and Society, Karolinska Institute, Stockholm, Sweden; 5 Theme Inflammation and Aging, Karolinska University Hospital, Huddinge, Sweden; 6 Research and Development Unit, Stockholms Sjukhem, Stockholm, Sweden; Gabriele d’Annunzio University of Chieti and Pescara: Universita degli Studi Gabriele d’Annunzio Chieti Pescara, ITALY

## Abstract

**Objective:**

To analyse if the health progression of geriatric Covid-19 survivors three months after an acute Covid-19 infection was worse than in other geriatric patients. Specifically, we wanted to see if we could see distinct health profiles in the flow of re-admitted Covid-19 patients compared to re-admitted non-Covid-19 controls.

**Design:**

Matched cohort study.

**Setting and participants:**

Electronic medical records of geriatric patients hospitalised in geriatric clinics in Stockholm, Sweden, between March 2020 and January 2022. Patients readmitted three months after initial admission were selected for the analysis and Covid-19 survivors (n = 895) were compared to age-sex-Charlson comorbidity index (CCI)-matched non-Covid-19 controls (n = 2685).

**Methods:**

We assessed using binary logistic and Cox regression if a previous Covid-19 infection could be a risk factor for worse health progression indicated by the CCI, hospital frailty risk score (HFRS), mortality and specific comorbidities.

**Results:**

The patients were mostly older than 75 years and, already at baseline, had typically multiple comorbidities. The Covid-19 patients with readmission had mostly had their acute-phase infection in the 1^st^ or 2^nd^ pandemic waves before the vaccinations. The Covid-19 patients did not have worse health after three months compared to the matched controls according to the CCI (odds ratio, OR[95% confidence interval, CI] = 1.12[0.94–1.34]), HFRS (OR[95%CI] = 1.05[0.87–1.26]), 6-months (hazard ratio, HR[95%CI] = 1.04[0.70–1.52]) and 1-year-mortality risk (HR[95%CI] = 0.89[0.71–1.10]), adjusted for age, sex and health at baseline (the CCI and HFRS).

**Conclusions and implications:**

The overall health progression of re-hospitalized geriatric Covid-19 survivors did not differ dramatically from other re-hospitalized geriatric patients with similar age, sex and health at baseline. Our results emphasize that Covid-19 was especially detrimental for geriatric patients in the acute-phase, but not in the later phase. Further studies including post-vaccination samples are needed.

## Introduction

Emerging evidence shows a wide range of systemic and organ-specific health problems weeks or months after an acute phase of Covid-19 (SARS-CoV-2) infection [1–3]. These health issues can be persistent and/or new conditions. After the acute phase, the quality of life is lower, and signs, symptoms, and diseases including fatigue, malaise, neurological, mental, cardiac, vascular, gastrointestinal, renal, musculo-skeletal, and dermatological disorders are more prevalent [1–3]. As older individuals have a higher risk for severe Covid-19 outcomes and the risk factors in acute Covid-19 differ by age [4–7], age group-specific analyses on health progression after Covid-19 infection are needed. This has been addressed by Cohen et al. (2022), and they reported the excess burden in multiple clinical conditions four months after initial Covid-19 infection in adults aged >65 years [8]. Another study with Covid-19 survivors aged >65 years by Liu et al (2022) reported 12% decrease in cognitive functions one year after Covid-19 hospitalization. Yet, the research evidence of the health progression after Covid-19 infection in the aged populations is limited and analyses in the most vulnerable and frail individuals with a high death rate from acute Covid-19 [9, 10], are needed.

In this study, we sought to analyse whether a previous acute Covid-19 infection is a risk factor for worse health progression three months after initial infection in hospitalized geriatric patients surviving at least three months. The health indicators were obtained from the electronic medical records including the admission with acute Covid-19 diagnosis and readmission after three months. In addition, we assessed whether the risk for new diseases was higher in Covid-19 patients compared to the controls. We hypothesised health progression to be worse and the risk for pathologies to be increased in the Covid-19 survivors.

## Methods

The analysis was performed in older patients with and without Covid-19 infection readmitted to geriatric hospitals (age at baseline at least 50 years). In the main analyses, we assessed the risk for worse health progression in the Covid-19 survivors compared to the matched non-Covid-19 controls using CCI≥2 (higher than the median (1)) (i), HFRS≥5 (intermediate or high risk of frailty)(ii), 6-months and 1-year-mortality (iii), and occurrence of new diseases (iv) as the outcomes.

### Clinical cohort

**Fig 1:**
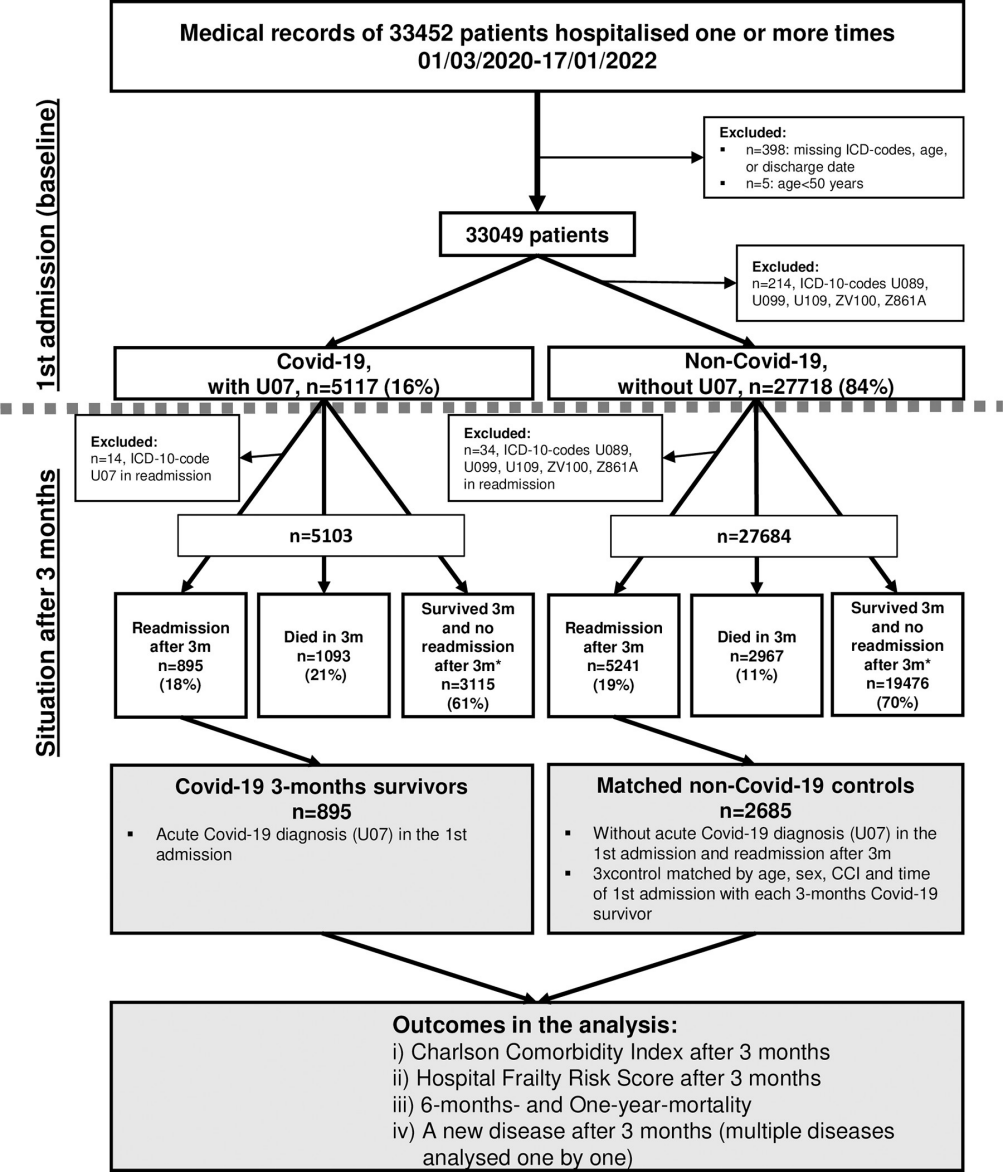
A flow chart showing the numbers of Covid-19 and non-Covid-19 patients in the records and the selection of the analytical samples for the main analysis. The analytical sample in the main analysis, the Covid-19 survivors and matched controls, is in grey boxes. Symbol ‘*’ indicates the groups of patients with no readmission to the nine geriatric clinics after 3 months. Abbreviations: CCI = Charlson comorbidity index, m = months.

All sample information used in the analysis were extracted from the electronic medical records and Death Register. International classification of diseases, 10th revision (ICD-10) system was used for the diagnoses. Selection and the timeline of the analytical sample for the main analysis are visualized in Fig 1. Data consisted of medical records of 33,452 patients admitted to nine geriatric hospitals in Stockholm, Sweden between March 1, 2020, and January 17, 2022. Of these, patients were excluded if: 1) data were missing (discharge date, diagnoses as ICD-codes or age, n = 398), 2) they were considered outliers, with age<50 years (n = 5), or 3) they had ICD-codes U089, U099, U109, ZV100 or Z861A in their 1^st^ admission records referring to post-infectious condition or history of Covid-19 disease (n = 214) (Fig 1) [11].

The 1^st^ admission was considered as the baseline in which 33,049 patients with and without acute Covid-19 diagnosis (U07) were divided into Covid-19 (n = 5,117, 16%) and non-Covid-19 (n = 27,718, 84%) patients, respectively (Fig 1). In our main analysis, only readmissions three months after the 1^st^ admission were considered, which is the time after acute and post-acute Covid-19 disease [12, 13]. Of the Covid-19 survivors with readmission after three months, 14 were excluded because they had an ICD-code U07 in their readmission records, and of the non-Covid-19 survivors, 34 patients were excluded because of ICD-codes U089, U099, U109, ZV100 or Z861A in their records referring to post-infectious condition or history of Covid-19 disease (Fig 1).

For the comparative main analysis, a case-control setting was assigned in patients with readmission data. For each Covid-19 survivor with readmission (n = 895, U07 only in the 1st admission), three age-sex-CCI-matched non-Covid-19 controls were selected out of the 5,241 non-Covid-19 patients, thus, resulting in a control group with 2,685 patients (Fig 1). Matching is described in paragraph, *Statistical analysis*.

### Covid-19 diagnosis

Acute Covid-19 disease was determined either by a positive laboratory result performed using transcriptase polymerase chain reaction (RT-PCR), or a clinical diagnosis for those with a negative RT-PCR but with typical symptoms and computed tomography scan findings (ICD-10-codes U07.1 or U07.2 [11]).

### Charlson comorbidity index, Hospital frailty risk score and mortality

In the analysis, a comorbidity measure (Charlson Comorbidity Index [CCI]) and a frailty measure (hospital frailty risk score [HFRS]) were based on ICD-codes. The CCI was computed using ICD-10-codes and weights as previously described [14]. The HFRS was defined from 109 ICD-10-codes that are overrepresented in the frail individuals [15]. The CCI and HFRS were calculated at baseline (in the 1^st^ admission) and after three months (in the 1^st^ readmission after three months).

Death dates between March 1, 2020, and January 17, 2022, were available for all 33,452 patients in the electronical medical records. We monitored if death occurred within 6 months or within one year after the 1^st^ admission to a hospital, and time to death for 6-months- and 1-year mortality was calculated from the 1^st^ admission date. Time between the 1^st^ admission date and end of data collection (17 January, 2022) was at least 6 months for 95% of 895 Covid-19 patients and 93% of 2,685 matched controls, and at least one year for 72% of 895 Covid-19 patients and 63% of 2,685 matched controls.

### Diseases

In the analysis of individual diseases, two approaches were used: a) all ICD-10-codes in hierarchical groups and b) selected diagnoses with specific ICD-codes. In the hierarchical approach (a), ICD-codes in the medical records in an admission were assigned into categories shown in Table 1 and Fig 3. The selected diseases (b) were *Pneumonia*, *Any fracture of fall*, *Malaise or fatigue*, *Heart failure*, *Stroke*, and *Dementia or confusion* (ICD-codes in Table 1). These selected diseases (b) were chosen for analysis as they are common conditions in the geriatric patients (i.e. a reasonable number of cases for statistical analysis) and/or have been previously suggested or shown to be one of the late effects of Covid-19 in younger and/or older adults [2, 8, 16, 17]. Occurrence of individual diseases (yes/no) for each patient were explored at baseline (in the 1^st^ admission) and after three months (in any readmission after three months until 17 January, 2022).

**Table 1:** ICD-codes for individual diseases.

**a.**	
**Disease category**	**ICD-codes**
AB: Infectious parasitic diseases	A00-B99
CD: Neoplasms including cancer	C00-D48
D: Diseases of the blood and blood forming organs and the immune mechanism	D50-D89
E: Endocrine, nutritional and metabolic diseases	E00-E90
F: Mental and behavioural disorders	F00-F99
G: Diseases of the nervous system	G00-G99
H-eye: Diseases of the eye and adnexa	H00-H59
H-ear: Diseases of the ear and mastoid process	H60-H95
I: Diseases of the circulatory system	I00-I99
J: Diseases of the respiratory system	J00-J99
K: Diseases of the digestive system	K00-K93
L: Diseases of the skin and subcutaneous tissue	L00-L99
M: Diseases of the musculoskeletal system and connective tissue	M00-M99
N: Diseases of the genitourinary system	N00-N99
R: Symptoms signs and abnormal clinical and laboratory findings not elsewhere classified	R00-R99
ST: Injury poisoning and certain other consequences of external causes	S00-T98
**b.**	
**Disease/condition**	**ICD-codes**
Pneumonia	J12-J18
Any fracture of fall	S12, S22, S32, S42, S52, S62, S72, S82, S92, M90, W00-W19
Malaise or fatigue	R539
Heart failure	I099, I110, I130, I132, I255, I420, I425, I426, I427, I428, I429, I43, I50
Stroke	G45, G46, H341, I60, I61, I63, I64
Dementia* or confusion	F00, F01, F02, F03, F051, G30, G31, A810, R41, F059, F051, F050

Two approaches were used: all ICD-10-codes in hierarchical groups (a) and selected diagnoses with specific ICD-codes (b).

*) Anti-dementia medication (ATC-codes N06DA or N06DX01) was also considered

### Statistical analysis

Control matching. The matching of the non-Covid-19 control patients with the 895 Covid-19 survivors was performed based on the 1^st^ admission data. Three controls were selected for each Covid-19 survivor and a control patient was required to have a readmission after three months, and to have the same sex, age quartile, CCI category (0,1 or 2+), and the 1^st^ admission inthe 1^st^ pandemic wave or after (cut point August 31, 2020). If more than three matches were found, of these, three controls were selected randomly.

Main analyses. Binary logistic regression was used to assess odds ratios (ORs) and corresponding CIs for the relationship between baseline Covid-19 diagnosis and the outcomes i, ii and iv. In the mortality analysis (iii), Cox regression was used to assess the hazard ratios (HRs) and corresponding CIs for the effect of the baseline Covid-19 diagnosis on the outcome.

All analyses (i-iv) were performed using the cross-validation in which 50 iterations were performed, each time randomly selecting 90% of the data. For each outcome, the cross-validated estimate (OR[i, ii, iv], HR[iii] and their corresponding CIs) was the mean of all estimates in the iterations. All models (i-iv) were adjusted for age and sex, as well as CCI (i), HFRS (ii) and CCI + HFRS (iv) at baseline. The analysis on individual diseases (iv) was performed using a negative-outcome-specific approach in which only patients without the disease in question at baseline were included. The health outcomes (i-iii) and individual diseases (iv) were analysed in separate models, one by one. All analyses were performed using R software (version 4.0.5). In the mortality analysis, R package survival (version 3.2–10) was used.

Additional analyses. Additionally, as a descriptive analysis, we visualized the high level of comorbidities in these geriatric patients using network presentation where node size corresponds to the prevalence of patients with the disease and the line that connects each node corresponds to the prevalence of co-occurrence of each disease pair. As the matched control group was three times larger than the Covid-19 patient group, node sizes and line widths were adjusted to be visually comparable between the groups by dividing the numbers of patients with a disease and co-occurrences of the diseases in the control group by the number 3. Networks were produced using R package igraph v1.2.9. Statistical significance (p<0.05) for the difference between baseline and readmission after 3 months within a patient group was assessed using Mann Whitney-U test for continuous and Fisher’s Exact Test for categorical variables.

### Ethics

This study was approved by the Swedish Ethical Review Authority (Dnr 2020–02146, 2020–03345, 2021–00595, 2021–02096). The data were de-identified prior to the analysis.

## Results

### Patient characteristics

Characteristics of the 3,580 patients used in the main analysis (895 Covid-19 3-months survivors and 2,685 age-sex-CCI-matched non-Covid-19 controls) are shown in Table 2, and S1 and S2 Tables. Of these patients, five out of six were more than 75 years old (lower quartile 78, median 84, and upper quartile 89 years) and 56% were women. Median time to readmission after the 1^st^ admission was 215 (lower quartile 135, upper quartile 326) in Covid-19 patients, and 206 (lower quartile 133, upper quartile 325) days in controls. The first Covid-19 vaccine was approved in Sweden 27 December 2020. Of the 895 Covid-19 patients with readmission after 3 months, 687 (77%) had their first admission before December 27, 2020.

**Table 2:** Characteristics of the Covid-19 3-months survivors and matched control patients in the main analysis.

		Covid-19, n = 895					Matched controls, n = 2685				
Variable		Baseline	After 3 months	p	6-months-follow-up	1-year-follow-up	Baseline	After 3 months	p	6-months-follow-up	1-year-follow-up
**Number of diseases**	**Median (mean)**	3 (3.5)	5 (4.9)	<0.001	-	-	4 (3.8)	5 (4.9)	<0.001	-	-
**CCI≥2**	**n(%)**	350(39.1)	439(49.1)	<0.001	-	-	1050(39.1)	1259(46.9)	<0.001	-	-
**HFRS≥5 **	**n(%)**	165(18)	260(29)	<0.001	-	-	667(25)	811(30)	<0.001	-	-
**Mortality**	**n(%)**	-	-	-	39(4)	120(13)	-	-	-	113(4)	381(14)

Additional information of the patients are shown in S1 and S2 Tables. p-value is for the difference between baseline and readmission after three months within a patient group (Mann Whitney U-test for Number of diseases, and Fisher’s test for categorical CCI≥2 and HFRS≥5). Times were calculated from the 1^st^ admission date (the baseline). Number of diseases was calculated using all ICD-10-codes in hierarchical disease groups. Abbreviations: CCI = Charlson Comorbidity Index, HFRS = Hospital Frailty Risk Score

**Fig 2:**
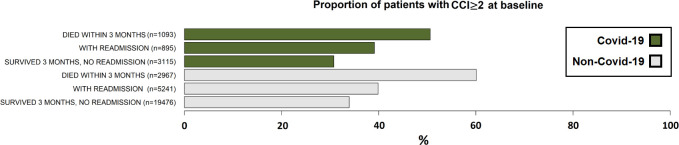
Geriatric patients with CCI≥2 at baseline (= the 1^st^ admission). Patients were categorised, as shown in Fig 1, based on acute Covid-19 infection in the 1^st^ admission, readmission after three months, and death within three months after the 1^st^ admission.

Characteristics of all geriatric patients in the 1^st^ admission (at baseline: Covid-19 n = 5,103, non-Covid-19 n = 27,684, Fig 1) are shown in S1 and S2 Tables. Of the 5,103 Covid-19 patients, 1,093 (21%) died within 3 months, 895 (18%) had readmission after three months, and 3,115 (61%) survived and had no readmission after three months (Fig 1). Of the 27,684 non-Covid-19 patients, 2,967 (11%) died within three months, 5,241 (19%) had a readmission, and 19,476 (70%) survived and had no readmission after three months (Fig 1). The proportions of patients with CCI≥2 at baseline within these patient groups are shown in Fig 2.

### Health status three months after acute Covid-19 infection

Patients in the main analysis had a statistically significant increase in the CCI, HFRS and number of diseases at readmission (Table 2). However, previous acute Covid-19 disease was not a risk factor for poorer health at readmission after three months compared to the matched controls when considering CCI (OR[95%CI] = 1.12[0.94–1.34], adjusted for age, sex and CCI at baseline) and HFRS (OR[95%CI] = 1.05[0.87–1.26], adjusted for age, sex and HFRS at baseline). Neither was previous Covid-19 disease a risk factor for 6-months (HR[95%CI] = 1.04[0.70–1.52] or 1-year mortality (HR[95%CI] = 0.89[0.71–1.10]), adjusted for age, sex, CCI and HFRS at baseline.

### Individual diseases

**Fig 3:**
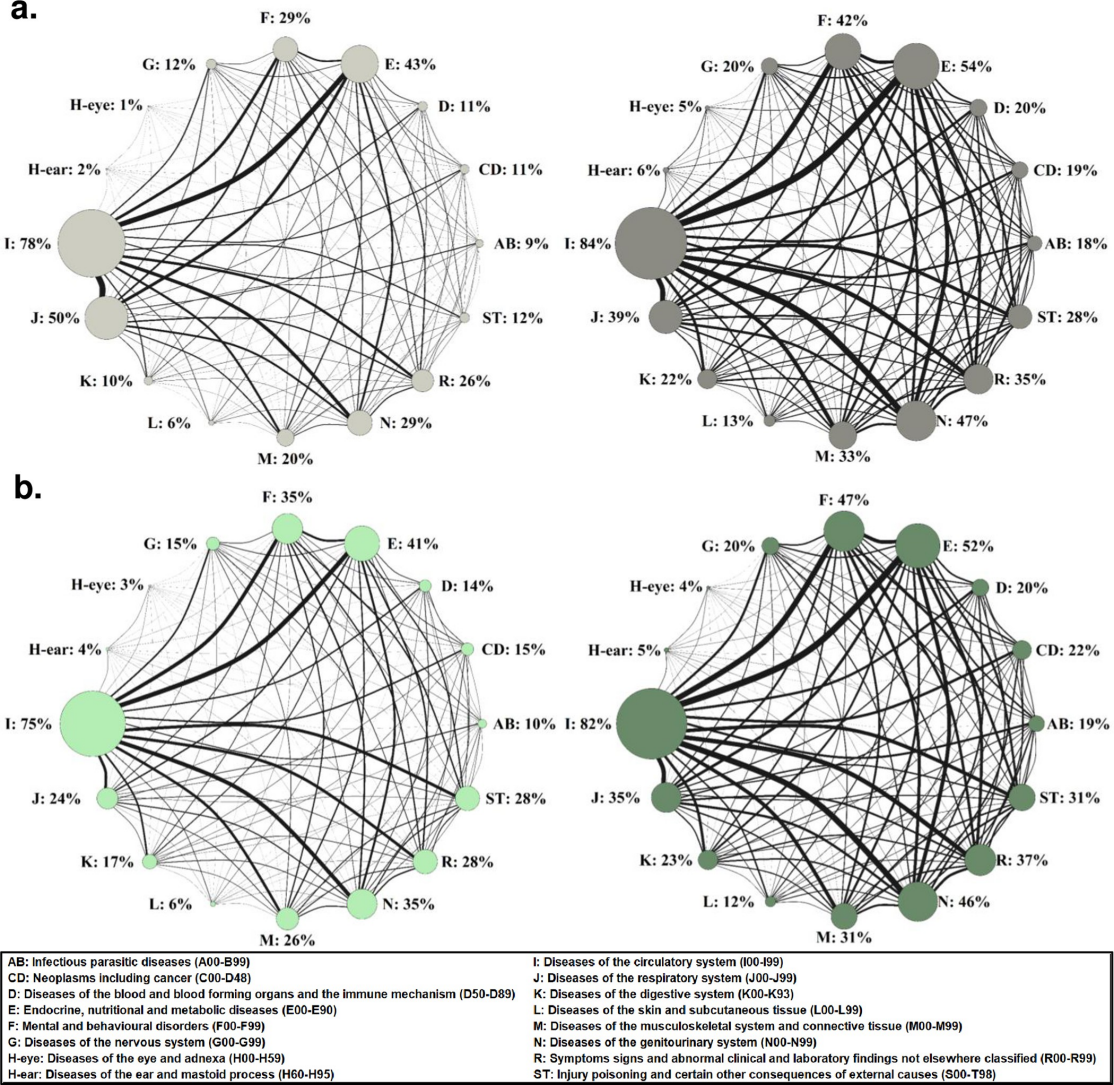
The prevalence of individual diseases and their co-occurrences in the 895 Covid-19 (a) and 2,685 matched control (b) patients in the 1^st^ admission and readmission after three months. Diseases in the 1^st^ admission are shown on the left, and in readmissions on the right side. The prevalence of the diseases are shown also in S3 Table. In the network presentation, node size corresponds to prevalence of a disease and the line that connects each node corresponds to prevalence of co-occurrence of each disease pair. As the control group was three times larger than the Covid-19 patient group, node sizes and line widths were adjusted to be visually comparable between the groups by dividing the numbers of patients with the disease and co-occurrences in the control group by the number, 3.

An overview of comorbidities in the geriatric patients at baseline and readmission after three months (S3 and S4 Tables) is shown in Fig 3. We found previous acute Covid-19 disease as a risk factor for ‘Diseases of the eye and adnexa’ (OR[95%CI]: 1.90[1.23–2.88]) and ‘Diseases of the ear and mastoid process’ (OR[95%CI]: 1.56[1.07–2.26]). The incidence rates for these diseases were 5% and 6% in the Covid-19 survivors versus 3% and 4% in the matched controls, respectively. Results for all analysed diseases are shown in Table 3A and 3B.

**Table 3:** New diseases at readmission in the Covid-19 survivors (n = 895) compared to the matched controls (n = 2,685).

**a.**	**Covid-19, n = 895**		**Matched controls n = 2685**			
**Outcome disease**	**Without the disease at baseline, n**	**New cases after 3 months, n(%)**	**Without the disease at baseline, n**	**New cases after 3 months, n(%)**	**OR**	**95% CI**
AB: Infectious, parasitic diseases	813	138(17)	2415	432(17.9)	0.93	0.74–1.15
CD: Neoplasms incl cancer	799	112(14)	2293	324(14.1)	0.99	0.77–1.26
D: Diseases of the blood and blood forming organs and the immune mechanism	795	131(16.5)	2310	360(15.6)	1.07	0.85–1.34
E: Endocrine nutritional and metabolic diseases	507	176(34.7)	1594	551(34.6)	1.01	0.81–1.26
F: Mental and behavioural disorders	637	172(27)	1746	517(29.6)	0.88	0.71–1.08
G: Diseases of the nervous system	788	105(13.3)	2277	278(12.2)	1.10	0.85–1.41
H-eye: Diseases of the eye and adnexa	882	40(4.5)	2616	65(2.5)	**1.90**	**1.23–2.88**
H-ear: Diseases of the ear and mastoid process	876	49(5.6)	2587	95(3.7)	**1.56**	**1.07–2.26**
I: Diseases of the circulatory system	199	128(64.3)	664	374(56.3)	1.40	1.00–1.99
J: Diseases of the respiratory system	448	95(21.2)	2029	465(22.9)	0.90	0.69–1.17
K: Diseases of the digestive system	805	159(19.8)	2229	426(19.1)	1.04	0.84–1.29
L: Diseases of the skin and subcutaneous tissue	840	95(11.3)	2529	269(10.6)	1.07	0.82–1.39
M: Diseases of the musculoskeletal system and connective tissue	717	171(23.8)	1990	430(21.6)	1.14	0.91–1.41
N: Diseases of the genitourinary system	634	234(36.9)	1752	583(33.3)	1.19	0.97–1.46
R: Symptoms signs and abnormal clinical and laboratory findings not elsewhere classified	665	219(32.9)	1928	634(32.9)	1.01	0.83–1.23
ST: Injury poisoning and certain other consequences of external causes	791	203(25.7)	1929	515(26.7)	0.95	0.77–1.15
**b.**	**Covid-19 n = 895**		**Non-Covid-19 controls n = 2685**			
**Outcome disease**	**Without the disease at baseline, n**	**New cases after 3 months, n(%)**	**Without the disease at baseline, n**	**New cases after 3 months, n(%)**	**OR**	**95% CI**
Dementia or confusion	739	94(12.7)	2187	291(13.3)	0.95	0.73–1.24
Pneumonia	651	70(10.8)	2563	227(8.9)	1.24	0.92–1.67
Any fracture or fall	831	117(14.1)	2256	334(14.8)	0.94	0.74–2.00
Malaise or fatigue	880	26(3)	2645	67(2.5)	1.19	0.72–1.91
Heart failure	671	151(22.5)	2060	394(19.1)	1.23	0.98–1.53
Stroke	877	35(4)	2574	91(3.5)	1.14	0.74–1.71

Diseases were based on either all ICD-codes in hierarchical groups (**a**) or more specific diseases (**b**). ORs (Odds Ratios) and 95% CIs (Confidence Intervals) adjusted for age and sex were calculated using logistic regression. Matched controls were used as a reference group for Covid-19 patients. ORs and 95% CI are bolded if p-value<0.05.

## Discussion

In this study, we explored in 3,580 geriatric hospitalised geriatric patients, mostly older than 75 years, the risk of previous acute Covid-19 infection for poorer health progression and specific diseases three months after the initial infection. As opposite to our hypothesis, the health progression of the Covid-19 survivors in this cohort did not differ from the matched control patients significantly. That is, we did not observe an association between previous acute Covid-19 infection and greater health decline indicated by the different health indicators (the CCI and HFRS) at the readmission, controlled for age, sex and the health at the baseline. Furthermore, previous acute Covid-19 disease was not a risk factor for a higher 6-months or 1-year-mortality among the 3-months survivors, adjusted for age, sex and health at the baseline. We based our initial hypothesis of Covid-19 being a clear risk factor for poorer health progression in these older hospitalised patients on the fact that homeostasis and resilience of the body lowers with advancing age [18, 19] and the fatality of acute Covid-19 disease has been the highest in the oldest ages [10, 20]. In addition, previous studies have shown a wide range of Covid-related long-term effects on health [1–3]. Age-related health risk factors including pre-existing comorbidities and frailty are suggested as likely contributors to more severe late effects of the Covid-19 infection after surviving from the acute phase of the disease [21]. However, extensive research evidence of these late effects in the geriatric hospitalised patients or nursing home residents has been lacking [22].

We also analysed previous acute Covid-19 infection as a risk factor for new individual diseases, adjusted for age and sex, and found out that the risk was significantly higher for two disease categories: ‘Diseases of the eye and adnexa’ and ‘Diseases of the ear and mastoid process’. Among the hierarchical disease categories, their incidence rates were the lowest in this geriatric population, less than six percent. For the other diseases than these two, the odds ratio was most often greater than one but not statistically significant. Hearing loss, visual disturbance and conjunctivitis are one of the late or persistent effects of Covid-19 infection (also referred as ‘post-Covid-19 syndrome’ or postacute sequelae of COVID-19, PASC) [2, 23, 24], and viral infections, in general, have manifestations in the eyes and ears [25, 26]. While in PASC, the late effects are often lingering symptoms for weeks or months after the initial infection, our disease-specific analysis focused on new conditions occurring after the acute Covid-19 infection. Mechanistically, Covid-19 virus can infect the inner ear cells directly [27], and possibly the conjunctival epithelium as well [28]. However, whether the location of initial infection has a role in the late pathophysiological consequences of Covid-19 infection remains to be disentangled, and in overall, the pathways leading to Covid-19-related late manifestations in the eyes and ears are still poorly characterised.

Previous studies focusing on the late effects of Covid-19 in individuals older than 65 years are limited. In the study by Cohen et al. (2022) with 90,000 older Covid-19 survivors, the incidence rates of multiple conditions in different organ systems were higher in the Covid-19 survivors compared to the ‘2020 controls’ or to ‘historical control group from 2019’. This clinical sequelae resembled in most parts those of viral lower respiratory tract illness. A major difference between the study by us and Cohen et al was that we did not exclude any individuals based on the health status while in the study by Cohen, exclusion was made if a person had some severe and/or disabling chronic conditions or was living in an institution. In addition, we used medical records from hospitalisations and general death registry data, while Cohen et al. used also other patient registries from outpatient hospital services.

We were expecting to see a clear difference in the health progression of the Covid-19 survivors compared to matched controls but this was not the case in the re-hospitalised geriatric patients. Instead, our data demonstrated the ageing-related health decline as well as a substantially high 3-months mortality rate in Covid-19 patients. Already at baseline, the patients with many diseases as indicated by the CCI and as visualised in the network diagrams in this report. Of the 33,000 patients initially hospitalised in these geriatric hospitals, one out of five died within three months if they had had acute Covid-19 infection and if not, one out of ten. Of the survivors, 20% had a readmission after three months, and according to the increased number of diseases, CCI and HFRS, the health of both Covid-19 and control patients was worse in the readmission than in the 1^st^ hospitalisation.

### Strengths and limitations

To the best of our knowledge, our study offers completely new information in the context of hospitalised geriatric patients. Primarily, we wanted to avoid exclusion of any patients and used comprehensive health indicators and disease diagnoses and mortality that were well available for analysis. Thus, data set in this study is representative of the whole population as it includes practically all patients hospitalized in these clinics in Stockholm, Sweden within the study period, between March 2020 and January 2022. Other advantages in the analysis were that we used as controls, non-Covid-19 patients hospitalized in the same clinics who were matched for age, sex and the health at the baseline. We were also able to use clinical data from several hospitals.

However, when interpreting our findings, some issues should be considered. First, the results cannot be generalised to all other older populations, such as community-dwelling older individuals. Health progression was evaluated only in geriatric patients with readmission to the nine geriatric hospitals and the CCI and HFRS after three months were not available for analysis for those patients who survived three months and were not readmitted to these clinics. Second, as we were able to use electronical medical records only, further research in the elderly is needed with questionnaire data targeted for Covid-19-related later symptoms. Third, in this analysis, most of the Covid-19 patients with readmission after three months had their 1^st^ admission during the 1^st^ two pandemic waves in which e.g. the prognosis of the geriatric Covid-19 patients was worse than in the later waves [29]. Therefore, Covid-19-related late effects need to be explored in geriatric patients with more later follow-up data. Fourth, many adverse health conditions in the oldest individuals may have increased during the pandemic also without Covid-19 infection because of restrictions and pressured health care services [30]. The recommendations in Sweden and other countries for individuals older than 70 years included limiting e.g. social contacts and being at crowded places. By decreasing daily activities and social interactions, cognitive and physical well-being are compromised and the risk for many conditions such as falls, dementia, and psychiatric symptoms increase [31, 32]. Historical data helps to explore whether the health profiles of geriatric patients in overall have changed during the pandemic. However, data before the pandemic were not available to our analysis. Lastly, due to sample size issue, we analysed individual diseases only in wide disease categories.

## Conclusions and implications

The health progression of Covid-19 3-months-survivors readmitted for geriatric care, did not differ dramatically from the other readmitted patients with similar age, sex and baseline health. Our results emphasize that Covid-19 was especially detrimental for geriatric patients in the acute-phase, but at least in this cohort not in the later phase. Future research is needed to assess the health progression after Covid-19 in the later pandemic waves.

## Supporting information

S1 Table Characteristics of all geriatric patients in the 1^st^ admission (baseline). Timeline and the grouping of the patients is described in Fig 1.
(DOCX)

S2 Table Descriptive of the health indicators in all geriatric patients. The CCI and HFRS are described in the 1^st^ admission (baseline) and a readmission after three months. Vital status was observed six months and one year after the 1^st^ admission. Timeline and the grouping of the patients are described in Fig 1.
(DOCX)

S3 Table Prevalence of individual diseases in geriatric 3-months Covid-19 survivors and matched non-Covid-19 controls in the 1^st^ admission (baseline) and readmissions after three months. Diseases were in hierarchical ICD-code categories.
(DOCX)

S4 Table Prevalence of individual diseases in geriatric 3-months Covid-19 survivors and matched non-Covid-19 controls in the 1^st^ admission (baseline) and readmissions after three months. Diseases were based on selected ICD-code-approach.
(DOCX)
