# Supplementary material for: Health progression for Covid-19 survivors hospitalized in geriatric clinics in Sweden
Source: PLoS One. 2023 Mar 22;18(3):e0283344. doi: 10.1371/journal.pone.0283344 (PMC10032538; doi:10.1371/journal.pone.0283344)
Supplement: S1 Table — Timeline and the grouping of the patients is described in Fig 1. (DOCX) [file pone.0283344.s001.docx]

S1 **Table.** C**haracteristics of all geriatric patients in the 1^st^ admission (baseline)**

Timeline and the grouping of the patients is described in Fig 1. Patient groups in the main analysis are highlighted with grey color.

|  |  | **Group size** | **Age** | **Men** | **Sum of admissions  during study period (3/2020-1/2022)** | **Days to the 1st**  **readmission after three months** | **The 1st admission date in time intervals*** | | | |
| --- | --- | --- | --- | --- | --- | --- | --- | --- | --- | --- |
| **Patient group** |  | **n(%)** | **Median (Q1, Q3)** | **n(%)** | **Median (min, max)** | **Median  (Q1, Q3)** | **1:  3/1/2020 -  31/8/2020 n(%)** | **2:  1/9/2021 -  31/1/2021 n(%)** | **3:**  **1/2/2021 -  20/4/2021 n(%)** | **4:**  **21/4/2021 -  17/1/2022**  **n(%)** |
| **Covid-19** | All | 5103(100) | 82(76,88) | 2412(47) | 1(1,16) | - | 1860(36.4) | 1820(35.7) | 820(16.1) | 603(11.8) |
|  | **Have readmission** | **895(18)** | **84(78,89)** | **391(44)** | **3(2,16)** | **215(135,326)** | **455(50.8)** | **322(36)** | **92(10.3)** | **26(2.9)** |
|  | Died within 3 months | 1093(21) | 86(81,91) | 580(53) | 1(1,4) | - | 494(45.2) | 408(37.3) | 115(10.5) | 76(7) |
|  | Survived 3 months,  no readmission | 3115(61) | 80(74,86) | 1441(46) | 1(1,7) | - | 911(29.2) | 1090(35) | 613(19.7) | 501(16.1) |
| **Non-Covid-19** | All | 27684(100) | 83(77,89) | 11175(40) | 1(1,22) | - | 7721(27.9) | 5482(19.8) | 2984(10.8) | 11497(41.5) |
|  | Have readmission | 5241(19) | 85(79,90) | 2085(40) | 3(2,22) | 200(132,314) | 2479(47.3) | 1385(26.4) | 640(12.2) | 737(14.1) |
|  | Died within 3 months | 2967(11) | 86(80,92) | 1451(49) | 1(1,6) | - | 1023(34.5) | 629(21.2) | 312(10.5) | 1003(33.8) |
|  | Survived 3 months,  no readmission | 19476(70) | 82(76,88) | 7639(39) | 1(1,6) | - | 4219(21.7) | 3468(17.8) | 2032(10.4) | 9757(50.1) |
|  | **Matched controls,  have readmission** | **2685(10)** | **84(78,89)** | **1173(44)** | **3(2,20)** | **206(133,325)** | **1365(50.8)** | **653(24.3)** | **319(11.9)** | **348(13)** |

*) as in (1)

**References**

1. Mak JKL, Eriksdotter M, Annetorp M, Kuja-Halkola R, Kananen L, Boström AM, et al. Two Years with COVID-19: The Electronic Frailty Index Identifies High-Risk Patients in the Stockholm GeroCovid Study. Gerontology. 2022 Nov 30;1–10.
