# Supplementary material for: Health progression for Covid-19 survivors hospitalized in geriatric clinics in Sweden
Source: PLoS One. 2023 Mar 22;18(3):e0283344. doi: 10.1371/journal.pone.0283344 (PMC10032538; doi:10.1371/journal.pone.0283344)
Supplement: S2 Table — The CCI and HFRS are described in the 1st admission (baseline) and a readmission after three months. Vital status was observed six months and one year after the 1st admission. Timeline and the grouping of the patients are described in Fig 1. (DOCX) [file pone.0283344.s002.docx]

S2 **Table.** **Descriptive of the health indicators in all geriatric patients**

The CCI and HFRS are described in the 1^st^ admission (baseline) and a readmission after three months. Vital status was observed six months and one year after the 1^st^ admission. Timeline and the grouping of the patients are described in Fig 1. Patient groups in the main analysis are highlighted with grey color.

|  |  | **Admission** | **Group size** | **CCI** | | **HFRS, n(%)** | | | **Mortality** | |
| --- | --- | --- | --- | --- | --- | --- | --- | --- | --- | --- |
| **Patient group** | |  | **n** | **Continuous**  **Average (median)** | **2 or higher**  **n(%)** | **Low risk:  <5**  **n(%)** | **Intermediate  risk: 5-15**  **n(%)** | **High risk:  >15**  **n(%)** | **6-months***    **n(%)** | **1-year***  **n(%)** |
| **Covid-19** | **All** | 1^st^ | 5103 | 1.4(1) | 1862(36.5) | 4035(79) | 1055(21) | 13(0.25) | 1238(24) | 1445(28) |
|  | **Have readmission** | **1^st^** | **895** | **1.5(1)** | **350(39.1)** | **730(82)** | **162(18)** | **3(0.34)** | **39(4)** | **120(13)** |
|  | **Have readmission** | **Readmission**** | **895** | **1.8(1)** | **439(49.1)** | **635(71)** | **256(29)** | **4(0.45)** | **-** | **-** |
|  | **Died in 3 months*** | 1^st^ | 1093 | 1.9(2) | 553(50.6) | 742(68) | 348(32) | 3(0.27) | - | - |
|  | **Survived 3 months*, no readmission** | 1^st^ | 3115 | 1.2(1) | 959(30.8) | 2563(82) | 545(17) | 7(0.22) | 106(3) | 232(7) |
| **Non-Covid-19** | **All** | 1^st^ | 27684 | 1.4(1) | 10473(37.8) | 20817(75) | 6755(24) | 112(0.4) | 4138(15) | 5318(19) |
|  | **Have readmission** | 1^st^ | 5241 | 1.5(1) | 2089(39.9) | 3979(76) | 1242(24) | 20(0.38) | 242(5) | 759(14) |
|  | **Have readmission** | Readmission** | 5241 | 1.8(1) | 2443(46.6) | 3664(70) | 1544(29) | 33(0.63) | - | - |
|  | **Died in 3 months*** | 1^st^ | 2967 | 2.5(2) | 1782(60.1) | 2050(69) | 902(30) | 15(0.51) | - | - |
|  | **Survived 3 months*, no readmission** | 1^st^ | 19476 | 1.3(1) | 6602(33.9) | 14788(76) | 4611(24) | 77(0.4) | 929(5) | 1592(8) |
|  | **Matched controls, have readmission** | **1^st^** | **2685** | **1.5(1)** | **1050(39.1)** | **2018(75)** | **653(24)** | **14(0.52)** | **113(4)** | **381(14)** |
|  | **Matched controls, have readmission** | **Readmission**** | **2685** | **1.8(1)** | **1259(46.9)** | **1874(70)** | **794(30)** | **17(0.63)** | **-** | **-** |

*) time calculated from the 1^st^ admission, **) the 1st readmission three months after the 1st admission
