# Supplementary material for: Health progression for Covid-19 survivors hospitalized in geriatric clinics in Sweden
Source: PLoS One. 2023 Mar 22;18(3):e0283344. doi: 10.1371/journal.pone.0283344 (PMC10032538; doi:10.1371/journal.pone.0283344)
Supplement: S3 Table — Diseases were in hierarchical ICD-code categories. (DOCX) [file pone.0283344.s003.docx]

S3 **Table.** **Prevalence of individual diseases in geriatric 3-months Covid-19 survivors and matched non-Covid-19 controls in the 1^st^ admission (baseline) and readmissions after three months**

Diseases were in hierarchical ICD-code categories.

|  | **Covid-19, n=895** | | **Matched controls, n=2685** | |
| --- | --- | --- | --- | --- |
| **Number of patients with disease (%)** | Baseline | After 3 months | Baseline | After 3 months |
| AB: Infectious, parasitic diseases | 82(9.2) | 157(17.5) | 288(10.7) | 487(18.1) |
| CD: Neoplasms incl cancer | 96(10.7) | 171(19.1) | 396(14.7) | 595(22.2) |
| D: Diseases of the blood and  blood forming organs and the immune mechanism | 100(11.2) | 180(20.1) | 381(14.2) | 543(20.2) |
| E: Endocrine nutritional and metabolic diseases | 388(43.4) | 481(53.7) | 1107(41.2) | 1428(53.2) |
| F: Mental and behavioural disorders | 258(28.8) | 378(42.2) | 933(34.7) | 1270(47.3) |
| G: Diseases of the nervous system | 107(12) | 175(19.6) | 421(15.7) | 558(20.8) |
| H-eye: Diseases of the eye and adnexa | 13(1.5) | 47(5.3) | 78(2.9) | 100(3.7) |
| H-ear: Diseases of the ear and mastoid process | 19(2.1) | 57(6.4) | 109(4.1) | 143(5.3) |
| I: Diseases of the circulatory system | 696(77.8) | 751(83.9) | 2018(75.2) | 2205(82.1) |
| J: Diseases of the respiratory system | 447(49.9) | 348(38.9) | 667(24.8) | 960(35.8) |
| K: Diseases of the digestive system | 90(10.1) | 200(22.3) | 446(16.6) | 603(22.5) |
| L: Diseases of the skin and subcutaneous tissue | 55(6.1) | 119(13.3) | 178(6.6) | 331(12.3) |
| M: Diseases of the musculoskeletal system and connective tissue | 178(19.9) | 291(32.5) | 700(26.1) | 794(29.6) |
| N: Diseases of the genitourinary system | 261(29.2) | 420(46.9) | 948(35.3) | 1198(44.6) |
| R: Symptoms signs and abnormal clinical and laboratory findings not elsewhere classified | 230(25.7) | 312(34.9) | 733(27.3) | 993(37) |
| ST: Injury poisoning and certain other consequences of external causes | 104(11.6) | 250(27.9) | 712(26.5) | 842(31.4) |
