# Supplementary material for: Health progression for Covid-19 survivors hospitalized in geriatric clinics in Sweden
Source: PLoS One. 2023 Mar 22;18(3):e0283344. doi: 10.1371/journal.pone.0283344 (PMC10032538; doi:10.1371/journal.pone.0283344)
Supplement: S4 Table — Diseases were based on selected ICD-code-approach. (DOCX) [file pone.0283344.s004.docx]

S4 **Table. Prevalence of individual diseases in geriatric 3-months Covid-19 survivors and matched non-Covid-19 controls in the 1^st^ admission (baseline) and readmissions after three months**

Diseases were based on selected ICD-code-approach.

| **Number of patients with disease (%)** | **Covid-19 n=895** | | **Matched controls n=2685** | |
| --- | --- | --- | --- | --- |
|  | Baseline | After 3 months | Baseline | After 3 months |
| Dementia or confusion | 156(17.4) | 216(24.1) | 498(18.5) | 698(26) |
| Pneumonia | 244(27.3) | 114(12.7) | 122(4.5) | 254(9.5) |
| Any fracture or fall | 64(7.2) | 136(15.2) | 429(16) | 442(16.5) |
| Malaise or fatique | 15(1.7) | 29(3.2) | 40(1.5) | 71(2.6) |
| Heart failure | 224(25) | 334(37.3) | 625(23.3) | 911(33.9) |
| Stroke | 18(2) | 36(4) | 111(4.1) | 111(4.1) |
